# Analysis of *Piscirickettsia salmonis* Metabolism Using Genome-Scale Reconstruction, Modeling, and Testing

**DOI:** 10.3389/fmicb.2017.02462

**Published:** 2017-12-11

**Authors:** María P. Cortés, Sebastián N. Mendoza, Dante Travisany, Alexis Gaete, Anne Siegel, Verónica Cambiazo, Alejandro Maass

**Affiliations:** ^1^Mathomics, Center for Mathematical Modeling, Universidad de Chile, Santiago, Chile; ^2^Facultad de Ingeniería y Ciencias, Universidad Adolfo Ibáñez, Santiago, Chile; ^3^Fondap Center for Genome Regulation (CGR), Santiago, Chile; ^4^Laboratorio de Bioinformática y Expresión Génica, Instituto de Nutrición y Tecnología de los Alimentos, Universidad de Chile, Santiago, Chile; ^5^DYLISS (INRIA–IRISA)–INRIA, CNRS UMR 6074, Université de Rennes 1, Rennes, France; ^6^Department of Mathematical Engineering, Universidad de Chile, Santiago, Chile

**Keywords:** pathogen, genome-scale, metabolism, constraint-based, *Piscirickettsia*, *salmonis*

## Abstract

*Piscirickettsia salmonis* is an intracellular bacterial fish pathogen that causes piscirickettsiosis, a disease with highly adverse impact in the Chilean salmon farming industry. The development of effective treatment and control methods for piscireckttsiosis is still a challenge. To meet it the number of studies on *P. salmonis* has grown in the last couple of years but many aspects of the pathogen’s biology are still poorly understood. Studies on its metabolism are scarce and only recently a metabolic model for reference strain LF-89 was developed. We present a new genome-scale model for *P. salmonis* LF-89 with more than twice as many genes as in the previous model and incorporating specific elements of the fish pathogen metabolism. Comparative analysis with models of different bacterial pathogens revealed a lower flexibility in *P. salmonis* metabolic network. Through constraint-based analysis, we determined essential metabolites required for its growth and showed that it can benefit from different carbon sources tested experimentally in new defined media. We also built an additional model for strain A1-15972, and together with an analysis of *P. salmonis* pangenome, we identified metabolic features that differentiate two main species clades. Both models constitute a knowledge-base for *P. salmonis* metabolism and can be used to guide the efficient culture of the pathogen and the identification of specific drug targets.

## Introduction

Piscirickettsiosis or Salmonid Rickettsial Septicaemia (SRS) is a severe systemic fish disease that targets multiple organs and tissues (Cvitanich et al., 1991). While it was first observed in the late 1980’s in Coho salmon cultured in farms on the south of Chile, outbreaks were later reported in different farming countries and in other salmonid fish species such as Atlantic salmon and Rainbow trout (Bravo, 1994; Cusack et al., 2002). SRS results in high mortality rates in farms, particularly in Chile, one of the main salmon producers, making it a great threat against the salmon aquaculture industry (Bustos, 2006). To date, the development of measures to control and treat this disease has had limited success (Henríquez et al., 2015) and depends heavily on the use of high dosages of antibiotics (Cabello, 2006).

The causing agent of SRS is *Piscirickettsia salmonis*, a fastidious facultative intracellular bacterium of the gamma-proteobacteria class. Over two decades ago, *P. salmonis* LF-89, the first known strain of this pathogen was isolated. Since then, studies on the fish pathogen have unveiled relevant clues, showing that it infects primarily macrophage cells and replicates inside intracellular vacuoles (McCarthy et al., 2008; Rojas et al., 2009; Ramírez et al., 2015).

Moreover, it has been recently reported that different *P. salmonis* strains are found along marine farms sites. In particular, it has been shown that two main phylogenetic clades, G1 and G2, can be identified among Chilean strains, both pathogenic, but each presenting different phenotypical features (Otterlei et al., 2016; Saavedra et al., 2017).

However, there are still several aspects of *P. salmonis* biology and pathogenicity that remain largely unknown, hindering the development of effective strategies against SRS (Rozas and Enríquez, 2014).

One key element that it is important to address is the metabolism of *P. salmonis*. It is known that pathogens adapt their metabolism to exploit available nutrients from host cells (Rohmer et al., 2011) and this adaptation goes hand in hand with virulence mechanisms that target host metabolic pathways (Kwaik and Bumann, 2013). Therefore, learning about pathogen metabolism is fundamental to understand bacterial pathogenesis.

A systematic way of studying an organism metabolism is through the development of a genome-scale metabolic model (GSM) and use of constraint based analysis (O’Brien et al., 2015). GSMs are generated starting with a detailed reconstruction of an organism metabolic network. These reconstructions are made based on annotated metabolic genes and proteins and are by themselves organism specific databases of their metabolic functions. Furthermore, GSMs are a mathematical representation of these reconstructed networks that include the addition of physicochemical and environmental constraints. Through constraint based analysis, these models can be used to predict *in silico* feasible metabolic phenotypes under different growth scenarios, as well as gene and metabolite essentialities (Lewis et al., 2012). The use of these models can thus drive hypothesis generation and guide experimental work.

GSMs have and continue to be developed for several pathogen organisms (Chavali et al., 2012). Main goals of these efforts have been to find links between metabolism and pathogenesis, to predict drug targets and in some cases to identify nutrient requirements (Chavali et al., 2008; Oberhardt et al., 2008). In the case of *P. salmonis* a first metabolic model for strain LF-89 was recently presented, accounting for 215 of the pathogen’s genes (Fuentealba et al., 2017).

Here we present iPS584, a new GSM for *P. salmonis* reference strain LF-89, a member of the phylogenetic clade G1 (Otterlei et al., 2016). The model includes 584 genes and additional pathways absent in the previous model. We explored *P. salmonis* metabolic network through the comparison of our model to those of other five bacterial pathogens in terms of their components and structure and highlight common and specific features. Additionally, through constraint-based analysis we predicted essential metabolites required by the fish pathogen and showed that a mixed diet of complementary carbon sources can benefit *P. salmonis* growth.

In addition, we constructed a model for strain A1-15972 (Bohle et al., 2014), member of clade G2 therefore covering two main species clades. Through the comparison of our models and analysis of *P. salmonis* pangenome we showed that particular metabolic genes not only set apart strains LF-89 and A1-15972 but also differentiate G1 and G2 clades because they are clade characteristic genes conserved in all of their strains.

## Materials and Methods

### Bacterial Strains, Media, and Growth Conditions

*Piscirickettsia salmonis* LF-89 (ATCC^®^ VR-1361) was obtained from the ATCC culture collection on 2013 and used to perform batch cultures. A *P. salmonis* LF-89 preculture was prepared by inoculating 50-ml Falcon tubes containing 5 ml of Austral SRS medium supplemented with 1 g/L Cysteine, 14 mL/L Fetal Bovine Serum, 30 g/L Casein-Peptone Soymeal-Peptone (CASO) Broth and 15 g/L sodium chloride. After approximately 7 days of cultivation, cells were extracted from the preculture and inoculated into 50 mL Falcon tubes containing 5 mL of a chemically defined medium to achieve an initial optical density at 600 nm (OD600) of approximately 0.1. The Falcon tubes were incubated at 18°C at a constant stirring of 160 rpm. OD600 was periodically measured. Experiments were performed in triplicate for five different defined media with different carbon sources. Briefly, Medium 1: Basal Medium (BM) containing glucose, amino acids and TCA compounds as carbon sources; Medium 2: BM without glucose; Medium 3: BM without TCA compounds; Medium 4: BM without glucose and TCA compounds; and Medium 5: Equal to Medium 4 but with higher concentration of glutamate (Full Media composition detailed in Supplementary Tables).

Additionally, growth cultures were performed for two additional *P. salmonis* strains (CRG01 and EM-90-like), one from clade G1 and one for clade G2. Clade membership was confirmed through a phylogenetic tree analysis (Supplementary Figure S5) using 16S rDNA sequences. The MAFFT tool (Katoh et al., 2017) was used for multiple alignment and tree construction and Phylo.io was used for tree visualization (Robinson et al., 2016). 16S rDNA sequences of 20 Piscirickettsia strains were downloaded from NCBI including *P. salmonis* CRG01 while for strain EM-90-like Sanger sequencing was performed (16S primers 27F (5′-AGAGTTTGATCCTGGCTCAG-3′) and 1492R (5′-CGGTTACCTTGTTACGACTT-3′).

Strains CRG01 and EM-90-like were cultivated in Media 3 and 4 using the same procedures used for strain LF-89 cultures.

### DNA Extractions, 16s rDNA Amplification, and PCR-RFLP

DNA was purified from samples of batch cultures at the beginning and end of the experiment, and then 16S rDNA was amplified using primers 27F/1292R. Amplified 16S rDNAs were digested using the restriction enzyme PmaCI and a 2% (w/v) agarose gel electrophoresis was performed to compare band patterns which were visualized in a UV transilluminator (Supplementary Figures S5, S6). The detailed protocol for DNA extraction and amplification as well as for PCR-RFLP are described in Mandakovic et al. (2016).

### Metabolic Network Reconstruction

*Piscirickettsia salmonis* LF-89 metabolic network reconstruction started with the generation of two automatic draft reconstructions. The first draft was created using Pantograph, an orthology-based method that uses as template a metabolic reconstruction of a related organism (Loira et al., 2015). We used *Escherichia coli* K-12 substr. MG1655 metabolic model iJO1366 (Orth et al., 2011) as a template. Orthologs between *E. coli* (RefSeq accession NC_000913) and *P. salmonis* (RefSeq accession NZ_CP011849.2) were determined using OrthoMCL v 1.4 and INPARANOID v 4.0. The second draft was generated using the PathoLogic component of Pathway-Tools software (Karp et al., 2002), taking as input *P. salmonis* LF-89 annotated genome (Pulgar et al., 2015b).

Drafts were merged and the result was manually assessed, including pathway and gene annotation curations. Additionally, all gene-reaction associations from transport reactions were re-evaluated based on gene annotations and BLAST searches against the Transport Classification Database TCDB. Network gap-filling was done using the meneco tool (Prigent et al., 2017) and the BiGG database as a source for additional reactions. This tool does topological gap-filling, searching for reactions whose incorporation in the draft model can complete paths from a set of seed metabolites to a set of target ones (Supplementary Tables).

The metabolic reconstruction of strain A1-15972 was made similarly, using as a template our LF-89 model and complementing it with the annotation of A1-15972 genome (RefSeq accession: NZ_ CP012413.1) which was built in the same way as that of strain LF-89, including blast searches against databases KEGG, NR, UNIPROT, and TCDB.

The genome-scale models for strains LF-89 and A1-15972 in sbml format are included in Supplementary Materials and they can also be downloaded from http://psalmonis.cmm.uchile.cl/models/.

### Metabolic Model Definition

#### Biomass Formulation

To represent biomass formation, we adapted the biomass function reaction from *E. coli* iJO1366 (Supplementary Tables). We kept the same main macromolecular components weight fractions. DNA composition was estimated from the GC content of *P. salmoni*s LF-89 genome; RNA composition was estimated based on *P. salmonis* LF-89 genome coding sequences; Protein composition was based on *P. salmonis* LF-89 predicted amino acid sequences; and *P. salmonis* LPS composition was taken from published experimental data (Vadovic et al., 2007; Vinogradov et al., 2013).

#### Constraint-Based Analysis

Flux Balance Analysis (FBA) was carried out using the python package COBRApy (Ebrahim et al., 2013) and the gurobi solver. Coupled and blocked reactions were determined using the flux coupling tool F2C2 (Larhlimi et al., 2012). To propose nutrients required for growth of *P. salmonis* the tool SASITA (Andrade et al., 2016) was used. Given a metabolic model, SASITA determines minimal sets of precursor compounds (*psets*) from a given universe of compounds required to produce a set of target compounds. The components of the biomass function were used as the target set and the universe of possible precursors was built including a variety of compounds such as sugars, fatty acids and the full set of amino acids in addition to cofactors and ions listed in Supplementary Tables.

All non-exchange reactions in the model were mass and charge balanced. Reaction reversibility information was taken from BiGG and MetaCyc databases. As common practice, reactions fluxes lower and upper bounds were set to –1000 and 1000 mmol/g DW/h for reversible reactions and to 0 and 1000 mmol/g DW/h for irreversible reactions.

*In silico* uptake rates for essential metabolites were left unconstrained (–1000 mmol/g DW/h flux lower bounds) for all FBA simulations. Main carbon uptake was limited to 6 mmol/gDW/h. Uptake rates of metabolites used as main carbon sources were constrained accordingly. Supplementary Tables contains the complete list of different *in silico* growth media used in FBA to simulate growth.

### Comparative Analysis

#### Gamma-Proteobacteria Pathogens Models

*Piscirickettsia salmonis* iPS584 metabolic model was compared with models iYL1228 (Liao et al., 2011), iPC815 (Charusanti et al., 2011), STM_v1.0 (Thiele et al., 2011), iMO1056 (Oberhardt et al., 2008), and iRS605 (Raghunathan et al., 2010) for the bacterial pathogens *Klebsiella pneumoniae* MGH 78578, *Yersinia pestis* CO92, *Salmonella Typhimurium* LT2, *Pseudomonas aeruginosa* PAO1, and *Francisella tularensis* LVS, respectively. All models were imported from the BiGG database with the exception of models iMO1056 and iRS605 retrieved from the BioModels database (IDs: MODEL1507180020 and MODEL1507180003). Ortholog groups between all pathogens were determined by OrthoMCL v1.4 using as input their corresponding protein sequences (NCBI accessions: CP000647.1, NC_003143.1, AE006468.1, AE004091.2, and AM233362.1).

#### Topological Comparison of Pathogens Metabolic Networks

We constructed metabolite graphs for model iPS584 and for each of the reference pathogen models mentioned above. These graphs are directed graphs where nodes represent metabolites in the model network. A directed edge between metabolites S and P was included in the graph if there is a reaction R in the network where S is a substrate and P is a product. In the case that R is reversible an edge from P to S was also incorporated in the graph. Transport reaction involving metabolites from different compartments were not included in the graphs.

The in-degree of a metabolite M in a graph corresponds to the total number of different metabolites S_i_ that are substrates in reactions where M is a product. Conversely, the out-degree of M is the sum of metabolites P_i_ that are products in reactions where M is a substrate (Supplementary Figures S1, S2).

To compare the connectivity of a metabolite in iPS584 with its connectivity in the reference pathogen networks we calculated its degree differences (in- and out-degree) between iPS584 and the average values for the reference networks. In this calculation, we only considered metabolites present in iPS584. The average degree value in the reference networks was calculated considering only the pathogen networks that also presented the metabolite.

#### *P. salmonis* Pangenome

All 19 complete genomes for *P. salmonis* strains available in the NCBI database were downloaded (Accession numbers in Supplementary Tables) and orthology among them was calculated with OrthoMCL v1.4. A binary matrix of ortholog gene groups versus strain was built and used to calculate a hamming distance between strains. Using these distances, a hierarchical clustering of *P. salmonis* strains was conducted to group strains.

## Results

### Overview of *P. salmonis* LF-89 Metabolic Model iPS584

The reconstructed metabolic network of *P. salmonis* iPS584 presented here, was generated by a combination of two complementary automatic approaches followed by thorough manual curation (details on Supplementary Material). A first draft reconstruction was generated using as a template *E. coli* iJO1366 metabolic model. We used this model because it is to date the most detailed metabolic reconstruction available for a gamma-proteobacteria. To enrich our first draft, a second *de novo* reconstruction was made based solely on the annotation of *P. salmonis* LF-89 genome, assuring in this way that all genes were considered. Both reconstructions were merged, and the result was manually evaluated and curated. In particular, reactions involved in lipopolysaccharide (LPS) biosynthesis inherited from *E. coli* were modified to be consistent with the reported lipid A carbohydrate backbone and fatty acid composition of *P. salmonis* (Vadovic et al., 2007; Vinogradov et al., 2013). Additionally, a biomass objective function was included in iPS584 for growth simulation purposes (Supplementary Tables). This function was derived and adapted from the *E. coli* iJO1366 model biomass function since the exact cellular composition of *P. salmonis* has not been determined. The LPS fraction in this objective function was filled by *P. salmonis* LPS macromolecule and the glycogen fraction was replaced by poly-3-hydroxybutanoate (PHB) as all three genes for its biosynthesis are in *P. salmonis* genome while no indication of glycogen biosynthesis was found.

**FIGURE 1:**
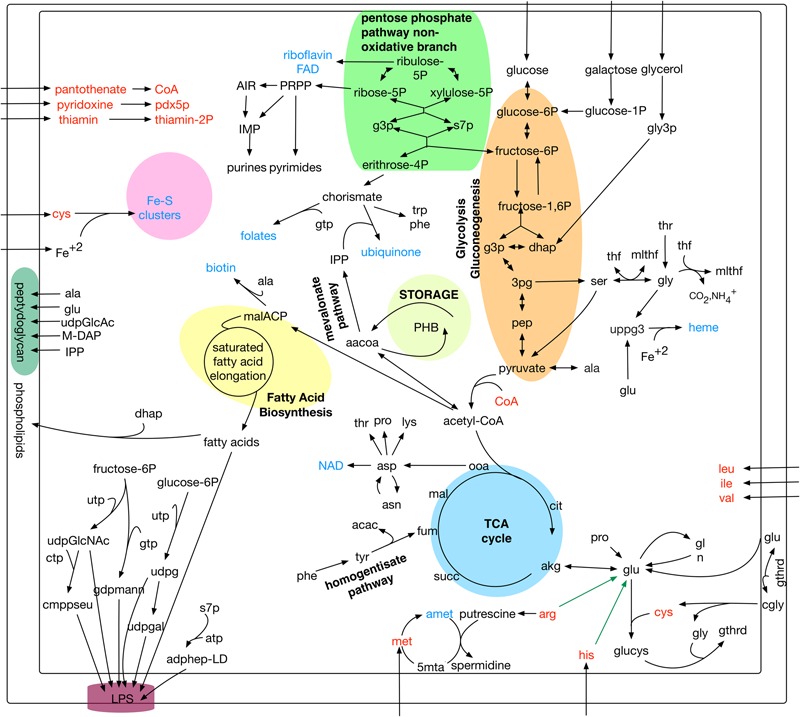
Overview of main metabolic pathways and reactions in *Piscirickettsia salmonis* LF-89 model iPS584. In red: amino acids and cofactors with incomplete or absent biosynthesis pathways. In blue: main additional cofactors included in iPS584 biomass function. Green arrows: pathways blocked in *pset* calculations: arginine and histidine degradation.

The iPS584 model contains 584 genes, accounting for 19% of genes in *P. salmonis* LF-89 genome; 801 unique metabolites and a total of 1,323 reactions. Among these reactions, 80% have a gene association. Reactions without a gene linked to them, are mostly transport or synthetic exchange reactions (257 in total). An overview of iPS584 is presented in **Figure 1**, while the full set of reactions, metabolites and genes in the reconstruction are listed in Supplementary Tables.

### Comparison of iPS584 and a Previous Model for *P. salmonis* LF-89

We compared iPS584 with model iPF215, the first metabolic model for *P. salmonis* LF-89 (Fuentealba et al., 2017). iPF215 allowed to predict that *P. salmonis* is auxotrophic for several amino acids and has the ability to use some of these compounds as carbon and energy sources. However, it contains only 445 metabolic reactions associated to 215 genes. This is a small number considering that there are a total 2,863 protein coding genes predicted in *P. salmonis* LF-89 genome. We found that 71% of iPF215 reactions are included in our iPS584 model (Supplementary Tables). Moreover, among these shared reactions, there are 30 reactions that were not linked to any gene in iPF215, but are now associated to genes in our model. We did not include in iPS584 the remaining reactions from iPF215 for either modeling differences (e.g., we modeled a reaction in a single step while it is in sub-steps in iPF215) or because we did not find enough evidence to support their incorporation. For example, reactions involving menaquinones and their biosynthesis form part of iPF215 even though no gene for their biosynthesis is present in the genome of strain LF-89. Additionally, we incorporated in iPS584 specific aspect of the pathogen that are absent in iPF215 apparently due to simplifications made in the biomass function that describes cell composition in this model. For example, we included biosynthesis pathways for PHB and for particular sugar nucleotides precursors of *P. salmonis* LPS. These pathways are not a part of iPF215 where it was assumed that *P. salmonis* stores glycogen and LPS composition was taken to be the same as in *Francisella tularensis*, which has a different sugar backbone to the one described for *P. salmonis* (Vadovic et al., 2007; Vinogradov et al., 2013).

### Comparison to Other Bacterial Pathogens Reconstructions

We analyzed the components and structure of *P. salmonis* LF-89 metabolic network through the comparison of our model to those of five other pathogens from the gamma-proteobacteria class (list in Section “Materials and Methods”).

#### Comparison of Pathogens Genomes and Genes

The size of *P. salmonis* reconstructed metabolic network is considerably smaller than that of most of the compared pathogens (Supplementary Tables). This can be largely explained by looking at the genome size of these pathogens, which, with the exception of *F. tularensis*, are between 40 and 82% larger than that of *P. salmonis*. This translates into a reduced number of genes and hence a potentially reduced metabolic capacity for the fish pathogen.

**FIGURE 2:**
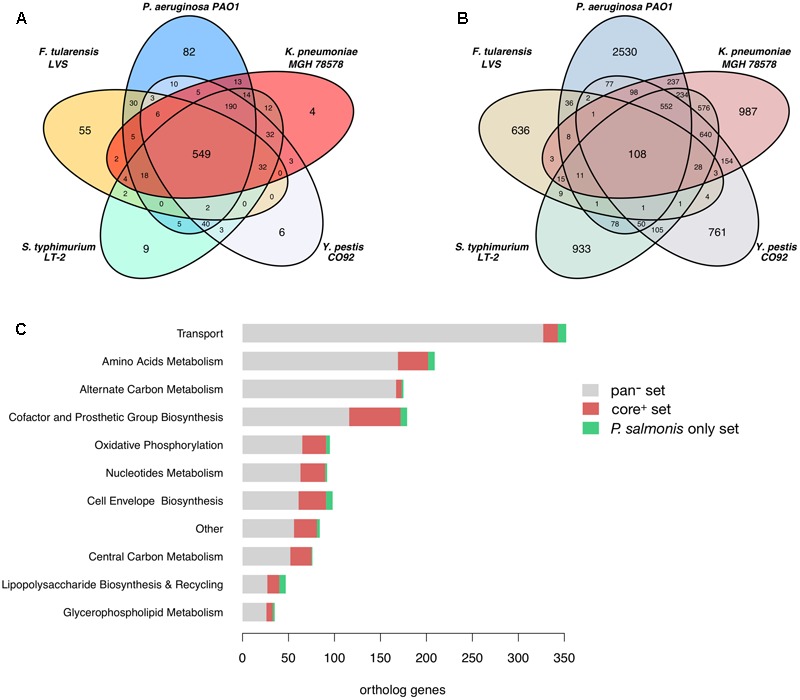
Ortholog genes among *P. salmonis* LF-89 and five other pathogen bacteria and links to their metabolic reconstructions. **(A)** Venn Diagram showing the distribution of *P. salmonis* LF-89 shared orthologs among the five other bacterial pathogens. **(B)** Venn Diagram of orthologs among the reference pathogens that are absent in *P. salmonis* LF-89. **(C)** Distribution among main metabolic categories of genes in the pathogens metabolic models: *pan^-^*: genes absent in *P. salmonis* LF-89; *core^+^*: core genes shared among all pathogens; *P. salmonis only*: genes only present in *P. salmonis* LF-89.

After an orthology analysis between all pathogens we found that *P. salmonis* LF-89 shares a total of 1,140 ortholog gene groups distributed among the compared pathogens as shown in **Figure 2A**. A core of 549 orthologs (core^+^ set) is shared between *P. salmonis* and all other five pathogens and 56% of them are accounted for in their metabolic models and are related to a wide range of metabolic functions (**Figure 2C**). They include 45% of the genes in *P. salmonis* iPS584 model. The remaining 8,879 gene groups from the five compared pathogens that do not have orthologs in *P. salmonis* LF-89 (pan^-^ set) are distributed as shown in **Figure 2B**. Most of them correspond to unique proteins in each pathogen and only 1,490 are part of their metabolic reconstructions. They are associated to reactions where the main categories are transport, amino acid and alternate carbon metabolism (**Figure 2C**). This suggest that these categories are either reduced in *P. salmonis* metabolic network or they are filled by reactions associated to alternative genes.

Among the 1,116 *P. salmonis* genes that are not shared with the compared bacterial pathogens, 59 are associated to reactions in the iPS584 reconstruction (**Figure 2C**). A large part of them corresponds to transporter genes, mostly putative peptide or amino acid transport systems. This correlates well with the inability of *P. salmonis* to produce several amino acids and the presence of several amino acid degradation pathways within its metabolic network (**Figure 1**). One of these pathways is the homogentisate pathway for the degradation of tyrosine and phenylalanine. This pathway is also present in *P. aeruginosa*, however, sequence similarity between the associated genes in *P. salmonis* and *P. aeruginosa* was insufficient to place them in the same ortholog groups.

Other noteworthy elements of *P. salmonis* metabolism included in iPS584 and associated to non-shared genes are the biosynthesis of CMP-pseudoaminate, the siderophore transport system ExbB-ExbD-TonB and a set of fatty acid desaturases.

CMP-pseudaminate is a sugar nucleotide precursor of pseudaminic acid, a particular residue found in *P. salmonis* LPS. The ExbB-ExbD-TonB siderophore transport system is a key element for the acquisition of iron from the host environment (Pulgar et al., 2015a). In the case of *P. salmonis* desaturases, one of the none-shared ones is similar to *Synechocystis* sp. PCC6803 DesC (39% identity) and an additional one is related to *P. aeruginosa* DesA (47% identity). Both are acyl-lipid desaturases that incorporate double bonds in membrane phospholipids (Murata and Wada, 1995; Kondakova et al., 2015). *P. salmonis* lacks the FabA-FabB system for the synthesis of unsaturated fatty acids, making desaturases a requirement for their production.

#### Comparison of the Topology of Pathogen Metabolic Networks

Another important difference between *P. salmonis* iPS584 and the compared pathogen metabolic networks is that iPS584 has a number of fully coupled reactions pairs (FC) that is 50% larger than the average FC pairs in the other networks. The presence of a high number of FC pairs could be an indication of missing reactions in the reconstruction or it could imply a lower flexibility of *P. salmonis* metabolic network associated to a smaller number of alternative pathways.

To explore this result, we further assessed the topological characteristics of *P. salmonis* iPS584 in relation to the reference networks. We evaluated the level of connectivity in each network by calculating their metabolites in- and out-degrees (see Materials and Methods). For each metabolite, we compared its degree values in iPS584 to its average values in the set of pathogen networks that also contain it (Supplementary Figure S3). In most cases, we found no differences, however, we could distinguish two sets of metabolites where differences were considerable: The *psal^-^* set, with metabolites much less connected in iPS584 with respect to the reference networks (degree values at least 4 units smaller) and a smaller set *psal^+^*, containing metabolites more connected in iPS584 (degree values 4 or more units larger). Metabolites from both sets are listed in Supplementary Tables.

For the less connected metabolites set *psal^-^*, we looked for reactions in the other pathogens networks that involve these metabolites and that are absent in iPS584. From these reactions, we identified the main metabolic pathways associated to them, present in most of the reference networks and either incomplete or missing in *P. salmonis*. We grouped these pathways into five main larger subsystems or categories listed in **Table 1**.

**Table 1:** Main metabolic pathways from reference pathogen networks that are associated to the less connected metabolites in iPS584 with respect to this reference and absent or incomplete in iPS584.

Subsystem	Missing or incomplete pathway in iPS584	Less connected metabolites in iPS584 (psal^-^)
Alternate carbon metabolism	Sugars, sugar phosphates, and sugar derivatives utilization	f6p, g3p, g1p, g6p, glc-D
	Glycolate and glyoxylate degradation	glyclt, glyc-R
	Threonine degradation to propanoate	ppa, ppcoa
Amino acids metabolism	Assimilatory sulfate reduction	so4, so3, grxox, grxrd
	L-cysteine biosynthesis	PAP, ac, cys-L
	L-methionine biosynthesis	cys-L
	L-ornithine biosynthesis	ac, acg5sa
	L-valine and L-leucine biosynthesis	3mob, 2obut
	L-histidine biosynthesis	his-L
Central metabolism	Citrate degradation	oaa
	Entner–Doudoroff pathway	g3p
	Glycogen biosynthesis and degradation	g1p
	Glyoxylate cycle	glx
	Oxidative Phosphorylation	ac, q8, q8h2, lac-D
	Pentose-Phosphate Pathway	g3p, g6p
Cofactor and prosthetic group biosynthesis	Menaquinol-8 biosynthesis	ichor
	Pyridoxal-5P biosynthesis	g3p
	Siroheme biosynthesis	ahcys
	Thiamine-PPi biosynthesis (thiazole moeity)	tyr-L, g3p, 4mpetz
Lipids and cell envelope metabolism	Cardiolipin biosynthesis	pg120, pg140, pg141, pg160, pg161, pg180, pg181, glyc[p], pg140[p], pg160[p], pg161[p], pg180[p], pg181[p]
	Cyclopropane fatty acid biosynthesis	ahcys
	Lipopolysaccharide (LPS) Biosynthesis /Recycling	updg
	Membrane phospholipid turnover	pg120, pg140, pg141, pg160, pg161, pg180, pg181, ddca, hdca, hdcea, ocdca, ocdcea, ttdca, ttdcea
Other	Purine degradation	glx
	cytidine and cytosine salvage	uri, ura, cytd
	Specific siderophores metabolism	udpg, fe3[e], fadh2, fmnh2, fad

*Metabolites in this table have either an in- or out-degree in iPS584 that is at least 4 units smaller than their average in the reference networks. All metabolites are cytoplasmic unless explicitly stated ([p]: periplasmic; [e]: extracellular).*

In the Amino Acids Metabolism subsystem, unsurprisingly, we find metabolites that are associated to several amino acid biosynthetic pathways in the compared networks but absent or incomplete in *P. salmonis.*

In the Cofactor and Prosthetic Group subsystem we find additional clues about the requirements for *P. salmonis* growth. The biosynthetic pathways of essential cofactors thiamine diphosphate and pyridoxal-5-phosphate are part of this category. In iPS584 glyceraldehyde 3-phosphate (g3p) out-degree is lower than in the rest of the pathogen networks because the enzyme 1-deoxyxylulose-5-phosphate synthase (DXS) is missing. DXS uses g3p and pyruvate to produce deoxyxylulose-5-phosphate and feed it to the aforementioned cofactors biosynthetic pathways. The absence of DXS indicates that *P. salmonis* requires the presence of these cofactors in its media for growth.

The Lipids and Cell Envelope Metabolism subsystem includes several less connected metabolites. This is mainly because *P. salmonis* lacks the enzymes required to synthesize cardiolipins and phosphatidylglycerols, and as a consequence several metabolites from these classes have lower in-degree in iPS584.

In the Alternate Carbon Metabolism subsystem, there are several metabolites that are less connected in iPS584 because *P. salmonis* lacks key enzymes needed for the degradation and utilization of several sugars and sugar derivatives. These include sugar phosphatases part of the degradation pathways of sugar phosphates, dehydrogenases and aldolases required to process sugar alcohols like mannitol or sorbitol, and sugars such as fucose or L-rhamnose and also sugar acids like galactonate or glucuronate. Additionally, several known genes for PTS transport systems used for the uptake of sugars are notably missing from *P. salmonis* genome. These observations indicate that these sugar compounds are unsuitable nutrients for *P. salmonis* and that this fish pathogen is adapted to a significantly different kind of diet than the compared pathogens.

We also found several metabolites from the Central Metabolism subsystem that are less connected in iPS584 because pathways from this category are missing or incomplete. Among them, we find the glyoxylate cycle, several oxidative phosphorylation redox reactions and also sugar catabolism related pathways including the oxidative branch of the pentose-phosphate pathway and the Entner–Dourdoroff pathway. The fact that central metabolism metabolites are less connected in *P. salmonis* network than in those of the compared pathogens shows that *P. salmonis* network is indeed less flexible because it possesses less alternative pathways to carry out central metabolic tasks.

The *psal^+^* set of metabolites with higher in- or out-degree in *P. salmonis* is made of only 9 metabolites (Supplementary Tables). The largest differences are found in Fe^+2^ and acetoacetyl-CoA. Fe^+2^ is more connected in iPS584 due to a difference in the modeling of the metabolic networks under comparison. We included the biosynthesis of Fe-S clusters in the metabolic reconstruction of *P. salmonis*, while for the other pathogens this pathway was omitted, making Fe^+2^ involved in a lower number of reactions. Acetoacetyl-CoA, on the other hand, has higher in- and out-degrees in iPS584 because it is involved in pathways absent in the other pathogens metabolic networks, namely the mevalonate pathway for isopentenyl diphosphate (IPP) biosynthesis and the biosynthesis and degradation pathways of poly-3-hydroxybutanoate (PHB). The metabolic tasks associated to these pathways are not unfulfilled in the compared pathogens networks but are accomplished using alternatives pathways. They synthesize IPP using the methylerythritol phosphate pathway and they can use glycogen as storage compound instead of PHB.

The smaller number of metabolites with higher degrees than with lower degrees with respect to the compared pathogens further indicates that *P. salmonis* metabolic network is considerable more rigid. Even by expanding the *psal^+^* set with additional metabolites more connected in *P. salmonis* network but to a lesser extent, it is still almost two thirds smaller than the set of least connected metabolites. Moreover, these additional metabolites are also associated to alternative or peripheral pathways like the mevalonate or the CMP-pseudaminate biosynthesis pathway.

### *In Silico* Analysis of *P. salmonis* LF-89 Nutrient Requirements

After observing this lower flexibility in its metabolic network, we explored what compounds could be exploited by *P. salmonis* LF-89 to fulfill its nutrients requirements. To do this, we analyzed different growth scenarios *in silico* using iPS584. We first defined what were the different minimal growth media that enable the *in silico* production of biomass.

This would give us an idea of which compounds are essential requirements and which are alternative nutritional sources for the pathogen. In order to do this, we calculated minimal precursor sets (*psets*) (Andrade et al., 2016). These are minimal sets of metabolites that when used in the *in silico* media of the model, they allow the production of biomass in FBA simulations. The removal of any metabolite of a *pset* results in a null growth rate.

We obtained two *psets* made of a total of 14 compounds including 12 that are essential metabolites found in both *psets* (**Table 2**). In line with our previous analysis of the network we found that these essential metabolites are amino acids and cofactors that compensate for incomplete or absent biosynthetic pathways. We also observed that differences among *psets* arise from the alternative use of glutathione and cysteine. Glutathione (γ-glutamyl-cysteinyl-glycine) can be used as cysteine source since it can be degraded through γ-glutamyl transpeptidase.

**Table 2:** Essential compounds for *in silico* growth of *Piscirickettsia salmonis* LF-89 and additional metabolites that can act as carbon and/or nitrogen source.

	Essential metabolites	Additional carbon and/or nitrogen sources
Amino acids	L-Isoleucine	Glycine
	L-Leucine	L-Alanine
	L- Valine	L-Asparagine
	L-Methionine	L-Aspartate
	L-Histidine	L-Glutamate
	L-arginine	L-Glutamine
	L-cysteine ^∗^	L-Phenylalanine
		L-Proline
		L-Serine
		L-Threonine
		L-Tyrosine
Cofactors and Vitamins	Pyridoxine	–
	Thiamine	
	D-pantothenate	
	Fe^+3^	
TCA intermediaries	–	Fumarate
		Malate
		Succinate
		Ketoglutarate
Sugars	–	D-Glucose
		D-Galactose
Other	Glutathione ^∗^	Glycerol
		Ammonium
		Adenine

*Amino acids are the only metabolites that can act as sources of both C and N, while adenine and ammonium are N sources only. ^∗^L-cysteine can be supplied directly or it can be obtained from glutathione.*

In addition to compensate for their auxotrophy, the amino acids L-histidine and L-arginine that make up the calculated *psets* also serve as carbon and nitrogen source because their degradation pathways can feed central metabolism. Since these metabolites are in themselves a requirement and *psets* are minimal, no other metabolites that could also feed central metabolism are included in these sets. To search for them, we did a second *pset* analysis, this time blocking in our model the degradation pathways of the mentioned amino acids (green arrows in **Figure 1**). As a result, we obtained 26 new sets (Supplementary Tables) where alternative metabolites listed in **Table 2** can act as carbon and/or nitrogen sources.

Carbon sources obtained through *pset* analysis enter central metabolism at different points. Sugars glucose and galactose enter at the beginning of glycolysis, while TCA intermediaries and amino acids enter at the level of the TCA cycle. Glycerol, on the other hand, enters central metabolism after being degraded to glycerone phosphate (dhap) which feeds both glycolysis and gluconeogenesis (**Figure 1**). Since central metabolism is fed at different levels by these nutrients, different pathways must be active for them to act as sole carbon sources. For sugars, glycolysis must be active, for TCA intermediaries and amino acids gluconeogenesis is a requirement for essential sugar compounds biosynthesis, while for glycerol both pathways must function.

**FIGURE 3:**
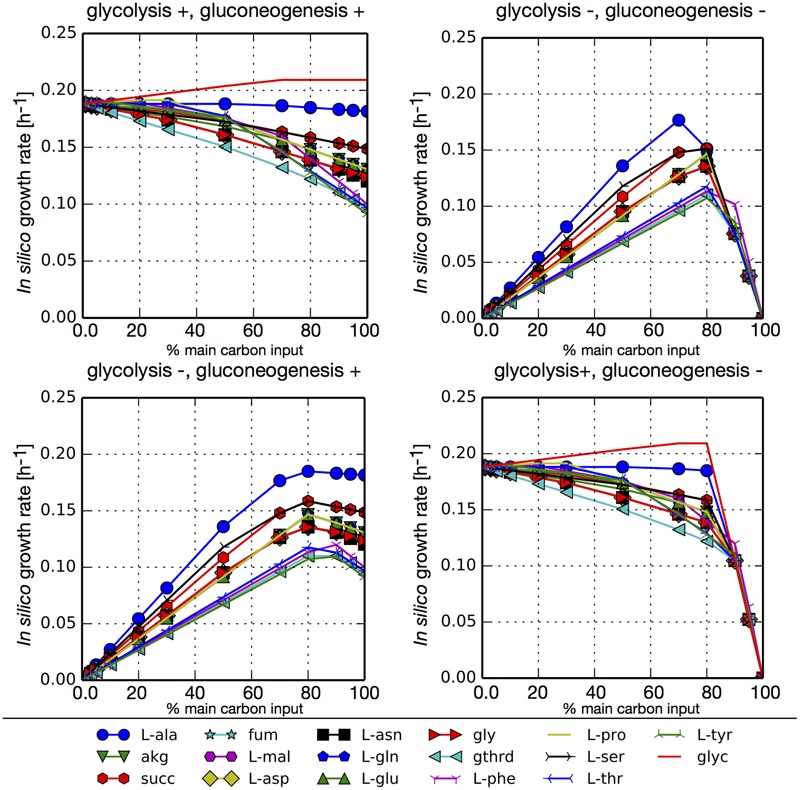
*In silico* growth rates under different growth scenarios for *P. salmonis* LF-89. FBA simulations were performed with model iPS584 using simultaneously two metabolites as main carbon input: glucose and an additional carbon source M (listed below the diagrams). In each simulation, maximum carbon uptake was limited to 6 mmol/gCDW-h and it was distributed between glucose and M in different ratios as depicted in each diagram (*x* axe) where the percentage of total carbon input flux associated to metabolite M is shown. For each (glucose, M) pair, four scenarios were analyzed corresponding to combinations of blocked/unblocked (+/–) gluconeogenesis or glycolysis. In all simulations, essential metabolites and ammonium uptake was unconstraint to avoid including additional limiting substrates.

To assess the use of the main carbon sources obtained through the previous *pset* analysis we simulated different growth scenarios. For each *in silico* media we evaluated growth with combinations of blocked or unblocked gluconeogenesis and glycolysis in our network. The main carbon input was arbitrarily limited to 6 mmol/gCDW-h and this flux was distributed between glucose uptake and an additional main carbon source identified from our *psets* (amino acid, TCA intermediary, or glycerol). Additionally, in each simulation cysteine and all essential metabolites found in all *psets* were allowed to freely enter the system. We also allowed the free uptake of ammonium so that nitrogen would not be a limiting substrate. **Figure 3** shows that all carbon sources can sustain the growth. In simulations where glycolysis was not blocked (glycolysis+), the largest growth rates are obtained when the highest fraction of carbon input comes from glucose, since its degradation through glycolysis generates additional ATP. The exception is glycerol, that also enters glycolysis but at a lower point and reaches slightly higher growth rates. Among the rest of the tested carbon sources, the highest growth rates were obtained for L-alanine, since it is transformed directly to pyruvate. Conversely, when glycolysis is blocked (glycolysis-), higher growth rates are obtained when most of the carbon that enters the network comes from TCA intermediaries or amino acids. Simulations with unblocked or blocked gluconeogenesis (gluconeogenesis+/–) did not result in major differences in growth rates since in both cases glucose was used for the synthesis of sugar compounds. The only significant difference is that in the case of an inactive gluconeogenesis (gluconeogenesis–) no growth is obtained without the addition of glucose.

We performed the same simulations without allowing ammonium to enter the network, therefore making amino acids the sole source of nitrogen (Supplementary Figure S4). This makes their input mandatory in order to obtain growth. In this scenarios, there is a trade-off between the need for C and N resources and so the highest growth rates are obtained when a combination of glucose and an amino acid is used. These are the points were the maximum amount of carbon would come from glucose without making nitrogen a limiting substrate.

#### *P. salmonis* LF-89 Growth on Defined Media

To study the growth of *P. salmonis* LF-89 under the main carbon sources identified through *pset* analysis, we constructed five different defined media and tested for growth in each of them (details in Section “Materials and Methods” and Supplementary Tables). Each media contained the essential metabolites previously mentioned and different sources of carbon. They were as follows: (1) a BM rich in carbon and energy sources made of the full set of standard amino acids, glucose and TCA intermediaries compounds; (2) BM medium without glucose; (3) BM medium without TCA compounds; (4) BM with no glucose nor TCA compounds; and (5) the same as the last medium but with a higher concentration of glutamate. **Table 3** shows the measured growth level (OD600) in each medium after 10 days of culture. We found growth was only achieved in media containing glucose pointing to an inactive gluconeogenesis. In particular, the use of a higher concentration of glutamate, could not compensate the absence of sugar in the medium. However, in the work of Fuentealba et al. (2017) growth was reported in a medium free of sugars where amino acids were used as sole carbon sources. This shows that gluconeogenesis is not necessary incomplete in *P. salmonis* LF-89 metabolic network.

**Table 3:** Measured OD600 for *P. salmonis* LF-89 batch cultures after 10 days of cultivation in five different chemically defined media.

Medium	OD600 (day 10)
1	1.26 ± 0.052
2	0.047 ± 0.016
3	1.22 ± 0.15
4	0.060 ± 0.013
5	0.018 ± 0.018

*Growth after 10 days was only observed for batch cultures on basal medium (BM) and BM without TCA intermediary compounds. Glucose was necessary to sustain growth and a supplement of L-glutamic acid was not able to restore growth. 1: BM; 2: BM without glucose; 3: BM without succinate, malate, fumarate, and ketoglutarate (TCA compounds); 4: BM without glucose and TCA compounds;5: BM with addition of glutamate and without glucose and TCA compounds.*

**FIGURE 4:**
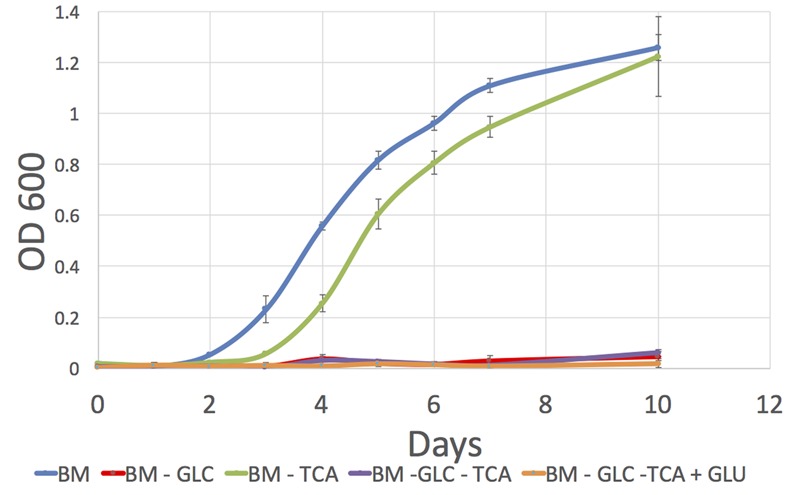
Growth curves for *P. salmonis* LF-89 on four different defined media. Media composition is listed in Supplementary Table S12. (1) BM, Basal Medium; (2) BM -GLC, Basal Medium without glucose; (3) BM-TCA, Basal Medium without succinic, malic, fumaric, and 2-oxoglutaric acids (TCA compounds); (4) BM-GLC-TCA, Basal Medium without glucose and TCA compounds; (5) BM-GLC-TCA+GLU, Basal Medium with addition of glutamate and without glucose and TCA compounds.

We obtained similar biomass levels in media (1) and (3) at the end of the culture but we observed different growth profiles with an earlier exponential phase start in media containing TCA intermediaries (**Figure 4**). This indicates that in addition to glucose, *P. salmonis* also profits from TCA compounds for growth as suggested by our *in silico* analysis.

### Analysis of Two *P. salmonis* Clades and Their Metabolic Features

We further evaluated our iPS584 model for strain LF-89 in the context of *P. salmonis* pangenome to determine how much was captured by the model at the levels of strain, clade, and genus. A gene orthology analysis between the 19 *P. salmonis* strains with an available complete genome assembly allowed us to clearly separate strains in two distinct groups, consistent with the previously described clades G1 and G2 (Supplementary Figure S9). Moreover, we found that the considered *P. salmonis* pangenome is made of a universe of 4,808 orthologs, 34% of them correspond to the core shared among all strains which includes most of the genes in iPS584 (Supplementary Figures S10, S11). We also found that no gene in iPS584 is particular to strain LF-89, however six genes in the model are only present in the genomes of clade G1 strains (**Table 4**). Among them is the gene for 5-aminolevulinate synthase, which allows these strains to produce heme precursor uroporphyrinogen-III from glycine. This gives their metabolic networks additional flexibility with respect to clade G2 strains which can only synthesize it using glutamate. Additionally, two of these clade G1 specific genes code for glycosyltransferases that we tentatively associated to the LPS formation reaction in iPS584. They are part of a large cluster of more than 10 genes related to cell wall polysaccharides biosynthesis present in all 19 *P. salmonis* strains. However, inside this cluster, two alternative groups of three genes differentiate clade G1 and G2 (Supplementary Figure S12). In all clade G1 strains, there is a group of genes coding for the same exact proteins: a protein of unknown function and the aforementioned glycosyltransferases. In clade G2 strains, these genes are replaced by a different set that codes for a membrane protein and two additional glycosyltransferases with low similarity to the ones from clade G1 (matches under 20% protein length and identity <30%).

**Table 4:** *Piscirickettsia salmonis* clade G1 and G2 specific genes in models iPS584 and iPSA590, respectively.

Gene ID	Protein ID	Description	Swissprot best hit	Best hit identity (%)
**clade G1**				
PSLF89_580	AKP72709.1	Putative homoserine efflux protein	O05406.2| YRHP_BACSU	30.1
PSLF89_04965	ALT18269.1	Nucleoside triphosphate pyrophosphohydrolase MazG	A0R3C4.1| MAZG_MYCS2	41.4
PSLF89_1179	AKP73168.1	Non-canonical purine NTP Phosphatase	Q8EIC6.1| NCPP_SHEON	57.5
PSLF89_1177	ALT18179.1	5-aminolevulinate synthase	P43089.2| HEM1_PARDP	48.3
PSLF89_3283	AKP74767.1	Glycosyltransferase	Q58577.1| Y1178_METJA	34.2
PSLF89_3284	AKP74768.1	Glycosyltransferase	D6Z995.1| MSHA_SEGRD	30.2
**clade G2**				
KW89_2115	ALA25581.1	Bifunctional acetaldehyde-CoA/alcohol dehydrogenase	P0A9Q8.2| ADHE_ECO57	70
KW89_2719	ALA26181.1	Aldehyde dehydrogenase	P33008.1| ALDH_PSESP	40
KW89_436	ALA23905.1	Tryptophan dioxygenase	Q1CVR6.1| T23O_MYXXD	29
KW89_869	ALA24337.1	dCTP deaminase	A4T0X9.1| DCD_MYCGI	32
KW89_2210	ALA25676.1	Cytidine deaminase	P19079.1| CDD_BACSU	35
KW89_2962	ALA26421.1	Uncharacterized 44.6 kDa Protein in region	Q48455.1| YC09_KLEPN	27
KW89_2963	ALA26422.1	Putative glycosyltransferase	Q914I4.1| GT048_SIFVH	31

Given these differences, we decided to build an additional metabolic model for clade G2 strain A1-15972, and therefore cover both clades. We used our iPS584 model as a template in addition to the annotation of A1-15972 genes. The resulting model iPSA590 is made of 1,291 reactions, 1,118 metabolites, and 590 genes (See Supplementary Tables). This model shares most of its reactions with iPS584 but it also incorporates additional reactions associated to clade G2 specific genes (**Table 4**). These genes include one for bifunctional alcohol/acetaldehyde dehydrogenase AdhE and one for an aldehyde dehydrogenase. These enzymes confer clade G2 strains the ability to produce ethanol from acetyl-CoA, a feature that is absent in G1 strains. Also among iPSA590 clade G2 particular features are cytidine and deoxycytidine deaminases, enzymes part of salvages pathways that allow the use of available cytidine and deoxycytidine as resources.

Regarding the use of different metabolites as carbon sources, as previously mentioned, models iPS584 and iPSA590 share most of their reactions since a large metabolic core is shared among *P. salmonis* strains. Thus, carbon sources identified *in silico* are the same for both models.

As described in the previous section, strain LF-89 cultures on different defined media only showed growth in cases were glucose was added and unexpectedly showed no growth on media containing amino acids as sole carbon source, in contrast with results obtained by Fuentealba et al. (2017). These differences led us to perform additional growth experiments using different *P. salmonis* strains. We used two available strains: an additional member of clade G1 (CRG01 strain) and a member of clade G2 (EM90-like strain). This clade assignation was confirmed through a phylogenetic analysis based on their 16S rDNA sequences (Supplementary Figure S5).

Growth cultures were carried on the previously described Media 3 and 4, i.e., one containing amino acids and glucose as carbon sources and one without glucose. Regardless of the clade, we observed similar growth patterns in all strains. Namely, for each strain growth was only obtained in media containing glucose, albeit different final biomass levels were observed in each case (Supplementary Figure S8) indicating that the observed phenotype is not particular of *P. salmonis* strain LF-89 and is also not clade dependent.

## Discussion

The two genome-scale models we presented here set a solid ground for metabolic studies on the intracellular fish pathogen *P. salmonis*. Model iPS584, for strain LF-89 was constructed combining automatic approaches and thorough manual curation, making an effort to incorporate all available information. It has over twice the genes and reactions in iPF215, the first metabolic model for the same strain, and incorporates relevant metabolic pathways and features absent in this previous reconstruction. We believe this constitutes a considerable improvement and shows that iPS584 is better suited to be the current knowledgebase of the pathogen’s metabolism.

A comparative analysis between iPS584 and the metabolic models of five other bacterial pathogens highlighted particular elements of *P. salmonis* metabolism that could be interesting to further explore with the aim to design drugs to fight piscirickettsiosis and to understand the links between metabolism and pathogenesis in the fish pathogen.

Desaturases seem to play a major role in *P. salmonis* lipid biosynthesis since, given the absence of the FabA–FabB system, they would be the only means for the pathogen to produce unsaturated fatty acids which are required for the formation of the cellular membrane. This suggests that desaturases could be interesting drug targets, considering in addition that a link between particular desaturases and virulence has been found in bacterial and yeast pathogens (Nguyen et al., 2011; Schweizer and Choi, 2011).

Lipopolysaccharide biosynthesis and export have been recognized as good targets for the design of antibiotics against gram negative bacterial pathogens (Whitfield and Trent, 2014). In iPS584, we included an LPS fraction as part of the biomass function. This makes the synthesis of these macromolecules and also of its precursors essential in our model. CMP-pseudaminate is a particular sugar nucleotide whose biosynthesis is required in the model to produce the pseudaminic acid residues part of *P. salmonis* LPS lipid A sugar backbone. Blocking CMP-pseudaminate biosynthesis in *P. salmonis* could also be an alternative strategy to fight this pathogen and it can benefit from previous work in *Helicobacter pylori*, where inhibitors of the enzymes that make this pathway were screened and selected (Ménard et al., 2014).

Another element that is relevant for pathogen survival is siderophore biosynthesis and transport, key for iron acquisition from the host environment (Skaar, 2010). We included among iPS584 reactions the ExbB-ExbD-TonB transport system, part of *P. salmonis* iron uptake mechanisms. However, even though *pvs* genes associated to siderophore biosynthesis have been identified in *P. salmonis*, the pathway has not been described in detail and could not be represented in iPS584. Thus, the link between siderophore biosynthesis and the rest of *P. salmonis* metabolic network is a knowledge gap that should to be address in future improvements of the model.

The comparisons of metabolic models at the topological level showed a lower flexibility in *P. salmonis* metabolic network. This is explained by the fact that there are metabolites less connected in the network of *P. salmonis* than in the compared networks and more importantly that some of them are associated with central metabolism reactions and pathways. Metabolites that are more connected in *P. salmonis* are much fewer and are associated with mostly peripheral pathways. Therefore, the additional connections in *P. salmonis* cannot make up for the absence of central metabolism components which can make the fish pathogen less adaptable to different environmental conditions and nutrient availability. This could be an important reason to explain why the laboratory cultivation of this bacterium is so difficult and why high growth rates are not achieved (Makrinos and Bowden, 2016).

Through constraint-based analysis we identified key metabolites required for *P. salmonis* growth. As has been previously shown, *P. salmonis* is auxotroph for several amino acids, including cysteine (Fuentealba et al., 2017). Our *in silico* analysis indicated that the requirement for cysteine could also be fulfill by glutathione, through the action of γ-glutamyl transpeptidase (GGT). Interestingly, this result was shown experimentally in a study in the closely related *Francisella tularensis*, which also requires cysteine for growth (Alkhuder et al., 2009). Moreover, it was demonstrated that the action of GGT is key for the intracellular growth of *F. tularensis* because glutathione is the main source of cysteine in the host cytosol. This way of adaptation to the host environment could also be a mechanism used by *P. salmonis*.

We also found that *P. salmonis* could exploit different types of carbon sources that feed the pathogen’s central metabolism at different levels. TCA intermediaries and amino acids that enter through the TCA cycle and sugars glucose and galactose as well as glycerol that feed glycolysis. Moreover, FBA simulations showed that it could growth through the simultaneous use of these different entry level substrates. This suggests that the pathogen has not adapted its metabolism for the use of a single type of resource.

We tested *P. salmonis* LF-89 growth by constructing different defined media using different combinations of the previously mentioned carbon sources. Growth was not observed in a media where amino acids were the only sources of carbon and energy. This was an unexpected result given the large battery of amino acid degrading pathways present in *P. salmonis* network and it is in contrast with a previous report of growth on an amino acid based media (Fuentealba et al., 2017).

We only observed growth in media containing glucose, which was also associated to higher growth rates in *in silico* simulations. Given these unexpected results, we performed similar growth experiments in two additional *P. salmonis* strains, one from the same phylogenetic clade as LF-89 (clade G1) and one from a different group (clade G2). We observed the same behavior that we observed for strain LF-89, that is, growth was obtained in the medium containing glucose and after 10 days, no growth was detected in the medium without the sugar. Notably, the same behavior was observed for the two additional strains. Even though we observed differences in final biomass concentration, all strains were able to grow in the medium containing glucose. We highlight that we verified the presence of *P. salmonis* and absence of contaminant agents in our cultures using 16S PCR-RFLP. Therefore, we confirmed the phenotypes observed for *P. salmonis*.

We believe that differences between results reported by Fuentealba et al. (2017) and ours could be associated to differences in culture conditions. Even though our culture conditions are similar in terms of carbon sources tested they do include differences, particularly in terms of salt compositions. One explanation could be that additional salts in our media could be impairing the uptake and degradation of some substrates. For example, in *E. coli* it has been shown that the presence of particular salts in the growth media can greatly limit glycerol fermentation due to enzyme inhibition as well as toxicity (Gonzalez et al., 2008). Another explanation for the differences in results could be related to the adaptation process performed in pre-cultures. While we grow the pre-culture of *P. salmonis* LF-89 in AUSTRAL-SRS, a complex medium containing a high concentration of glucose (Yañez et al., 2012), Fuentealba et al. (2017) grew the bacteria in a complex medium rich in amino acids and peptides (Henríquez et al., 2013) which could explain why they observe growth on their defined media.

Additionally, LF-89 strain growth profile differences between media with and without TCA intermediary compounds indicated that these substrates were also exploited by the fish pathogen. In other bacterial intracellular pathogens, such as legionella or chlamydia, an adaptation to a co-substrate diet has been observed (Häuslein et al., 2016; Mehlitz et al., 2017). Moreover, in the case of legionella it has been shown that different life-stages are associated to different substrate use (Eisenreich and Heuner, 2016). Metabolic models could be used to explore this possibility in *P. salmonis*, for example to analyze what pathways could be active in different stages. In order to do this, additional information should be incorporated into the models to accurately describe these different scenarios. For instance, different biomass functions describing the cellular composition of each stage should be included.

We broaden our study of *P. salmonis* metabolism through the analysis of its pangenome. This showed that most genes from model iPS584 for strain LF-89 are conserved and found in all 19 analyzed strains, which is consistent with pangenome analysis of other bacterial species where the majority of metabolic genes have been found to be core elements that constitute a species metabolic backbone (Islam et al., 2010; Monk et al., 2013; Bosi et al., 2016).

Additionally, we found that most non-core genes in iPS584 were not specific for strain LF-89 but characteristic of the genotypic clade G1 of which it is member. Furthermore, we constructed iPSA590, an additional genome-scale model for strain A1-15972, member of clade G2. Analysis of this model lead us to the same result, specific non-core genes in the model were conserved among G2 strains. This shows that small metabolic traits can differentiate these clades.

A particularly interesting differentiating feature is the bifunctional alcohol/acetaldehyde dehydrogenase AdhE whose coding gene is present in G2 strains only. It has been shown that AdhE controls virulence genes in *E. coli* O157:H7 by directing the flux from the acetyl-CoA pool and avoiding intracellular acetate accumulation (Beckham et al., 2014). Even though the effect of AdhE in that study was seen on type Three Secretion System (T3SS) which appears to be absent in *P. salmonis*, the difference on AdhE presence between clades could still suggest that alternative virulence regulation mechanisms related to metabolism are in play.

Further analysis could show if the metabolic particularities of G1 and G2 clades included in models iPS584 and iPSA590 can be linked to clade phenotypic differences such as those previously observed related to antibiotic susceptibility, particular *in vitro* growth profiles and host preferences (Otterlei et al., 2016; Saavedra et al., 2017).

## Author Contributions

MC carried out metabolic reconstructions, model definitions, constraint based analysis, and model comparisons. SM and AG designed and carried out all growth experiments. DT performed pangenome orthology analysis and genome annotations. AM and VC conceived the project and coordinated and oversaw its progress with AS. MC wrote the manuscript with critical revisions from AS, AM, and VC. All authors read and approved the final manuscript.

## Conflict of Interest Statement

The authors declare that the research was conducted in the absence of any commercial or financial relationships that could be construed as a potential conflict of interest. The handling Editor declared a shared affiliation, though no other collaboration, with one of the authors, AS.
