# Supplementary material for: Analysis of Piscirickettsia salmonis Metabolism Using Genome-Scale Reconstruction, Modeling, and Testing
Source: Front Microbiol. 2017 Dec 11;8:2462. doi: 10.3389/fmicb.2017.02462 (PMC5732189; doi:10.3389/fmicb.2017.02462)
Supplement: Supplementary file 1 [file Table_1.DOCX]

Supplementary Material:

Analysis of *Piscirickettsia salmonis* Metabolism Using Genome-Scale Reconstruction, Modeling and Testing

María Paz Cortés^*^, Sebastián Mendoza, Dante Travisany, Alexis Gaete, Anne Siegel, Verónica Cambiazo, Alejandro Maass

*** Correspondence:** María Paz Cortés: mpcortes@dim.uchile.cl

# Metabolic networks reconstruction details

## Reconstruction of *P. salmonis* LF-89

*Piscirickettsia salmonis* LF-89 metabolic reconstruction started with the generation of two automatic draft reconstructions made using two different tools: Pantograph and PathwayTools. Pantograph creates drafts based on gene orthology with a close organism whose metabolic network was previously reconstructed. PathwayTools, on the other hand, uses the genome annotation of the studied species to generate a draft metabolic network.

These two initial drafts were later merged and manually curated. Results obtained with both tools are summarized in Supplementary Table 1.

**Supplementary Table 1:** Number of reactions obtained in each automatic draft reconstruction of *P. salmonis* LF-89 metabolic network.

|  | **Orthology based draft (Pantograph)** | **Annotation based draft (Pathologic)** |
| --- | --- | --- |
| Reactions | 1229 | 1508 |
| Gene associated reactions | 769 | 1084 |
| Kept reactions in iPS584 | 871 | 561 |
| Kept common reactions | 446 | |

Details on the main steps of the reconstruction follow.

### Draft based on orthology

Pantograph takes as input a template model from a reference species and the list of orthologs between the template and the studied species. We used *E. coli* K-12 MG1655 and its iJO1366 metabolic model as reference.

The generated draft includes all reactions from the template for which there are orthologs genes in the studied species that can support its gene association, as stated in the template model. For example, if a reaction A in the template is associated to genes 1 *and* 2 and only gene 1 has an ortholog in the studied species, the reaction is not incorporated in the draft. However, if the reaction is associated to gene 1 *or* gene 2, then it is imported to the draft reconstruction associated to the ortholog of gene 1. Reactions without gene associations in the template are also incorporated in the draft.

### Draft based on annotation

PathwayTools software Pathologic component allows for the automatic generation of Pathway/Genome Databases (reconstructions) based on the annotated genome of an organism and a reference pathway database. These reconstructions are based on a set of predefined pathways in the MetaCyc database, if there is enough evidence for these pathways (associated genes) they are fully included in the reconstruction. This can lead to the incorporation of reactions without gene associations corresponding to pathway gap-fills. Manual steps can be followed within Pathologic to improve the reconstruction, however for *P. salmoni*s LF-89 draft, we only performed the software automatic steps. Manual curation was later performed outside PathwayTools.

### Merging drafts and manual curation

Reactions from each draft were reviewed. From the orthology based draft 354 reactions were removed: 279 without gene associations, mostly transport and exchange reactions and 75 with gene associations because after assessment of the annotations of their associated genes it was determined that they were miss assigned. Additionally, the gene association of 63 of the 871 kept reactions were manually modified, mostly by including additional associated genes based on their annotation.

In the case of the draft made with Pathologic, 561 reactions were kept and the rest was removed. Eliminated reactions included reactions without gene association, reactions related to the metabolism of macromolecules such as DNA or RNA not included in the model and reactions associated to generic E.C numbers.

After reaction removal, both drafts were manually merged. Metabolites and reactions identifiers (IDs) in the first draft were IDs from the BiGG database, while the second draft had MetaCyc IDs. In order to merge both drafts we translated MetaCyc to BiGG IDs. This translation was done using crosslink information available in both databases and in MetaNetX that was later manually checked to include missing matches and delete erroneous ones. We kept BiGG reaction and metabolites IDs when possible; MetaCyc IDs for elements outside BiGG and new IDs for those absent in both databases.

We obtained a total of 986 reactions, 446 common to both drafts, 425 exclusively from the orthology based draft and 115 unique to the annotation draft related to pathways and reactions in *P. salmonis* and absent in *E. coli* network reconstruction such as CMP-pseudaminate and PHB biosynthesis pathways.

Additionally, after manual curation, several transport and exchange reactions were added to the model. Genes associated to transport reactions are listed in Supplementary File 2 with their description and TCDB database best match. In cases were no specific transporter was found but the corresponding metabolites were essential requirements transport reactions were gap-filled.

Main additional reactions included in manual curation are fatty acid desaturases reactions and reactions for *P. salmonis* LPS biosynthesis.
