# Supplementary material for: Analysis of Piscirickettsia salmonis Metabolism Using Genome-Scale Reconstruction, Modeling, and Testing
Source: Front Microbiol. 2017 Dec 11;8:2462. doi: 10.3389/fmicb.2017.02462 (PMC5732189; doi:10.3389/fmicb.2017.02462)
Supplement: Supplementary file 4 [file Image_1.PDF]

Supplementary Material:

Analysis of *Piscirickettsia salmonis* Metabolism Using Genome-Scale Reconstruction, Modeling and Testing

María Paz Cortés*, **Sebastián Mendoza, Dante Travisany, Alexis Gaete, Anne Siegel, Verónica Cambiazo, Alejandro Maass**

*** Correspondence:** María Paz Cortés: mpcortes@dim.uchile.cl

1 Supplementary Figures

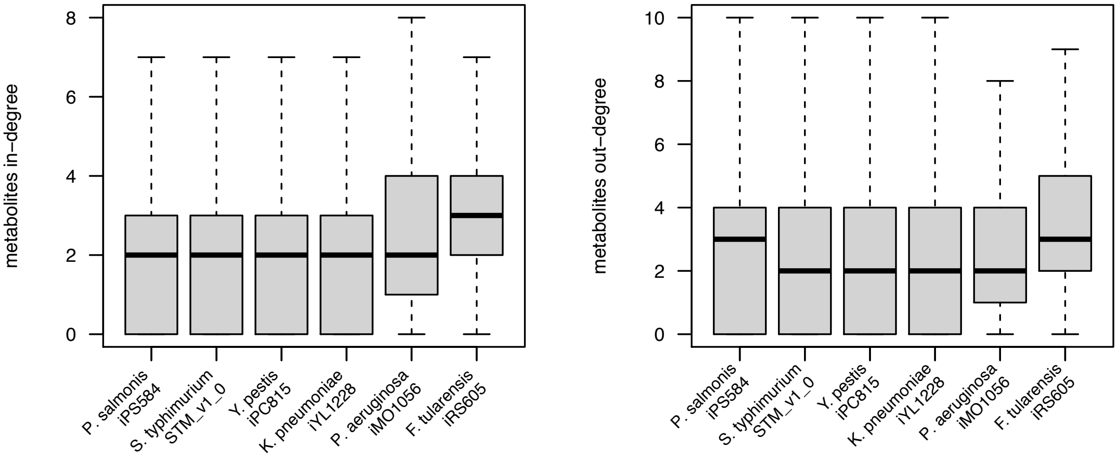

Supplementary Figure 1: Metabolite in- and out-degrees in the reconstructed metabolic networks of *P. salmonis* LF-89 and five other gamma-proteobacterial pathogens. A metabolite M in-degree was calculated as the number of different *s* metabolites that are substrates of a reaction in N where M is a product, while its out-degree corresponds to the number of P_i metabolites that are products in reactions were M is a substrate. Transport reactions between compartments were not considered for metabolite degree calculations.

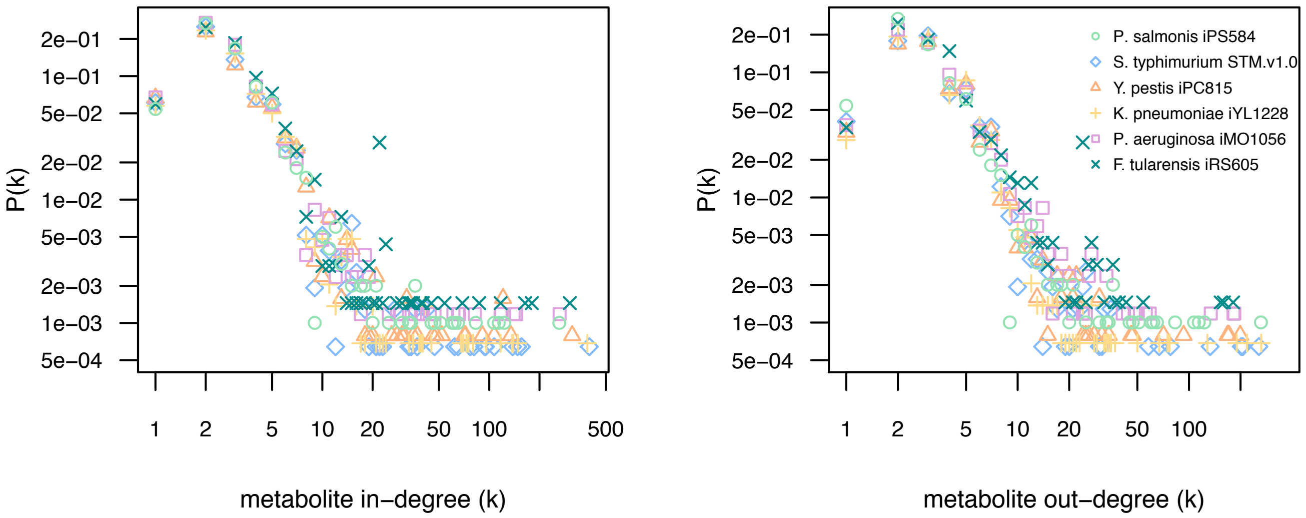

Supplementary Figure 2: Metabolite degree distribution P(k) in the metabolic networks of *P. salmonis* LF-89 and five other bacterial pathogen networks. P(k) corresponds to the fraction of metabolites with degree k in a network N. Left in-degree distribution. Right: out-degree distribution. In all cases, the degree distribution characteristic of metabolic networks is observed: The majority of metabolites in a network are weekly connected evidenced by a small in- and out-degree, while a very small number of them has a large number of links, indicating their presence in a large number of reactions

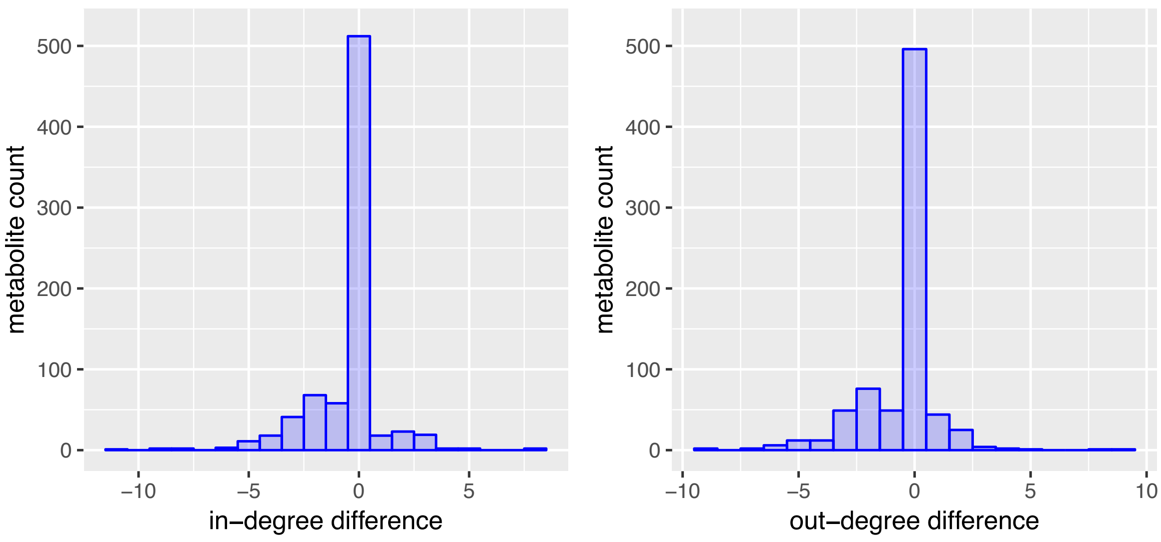

Supplementary Figure 3: Histograms of metabolite connectivity differences between *P. salmonis* iPS584 and the compared pathogen metabolic networks. For each metabolite in iPS584, we calculated the difference in degree values between iPS584 and the average degree in the compared networks that included it. Metabolites with differences > 0 have a higher degree in iPS584, while metabolites with values < 0 are less connected in iPS584 with respect to the compared networks. Hub metabolites with average in or out-degree higher that 20 were not considered. Left: In-degree differences. Right: Out-degree differences.

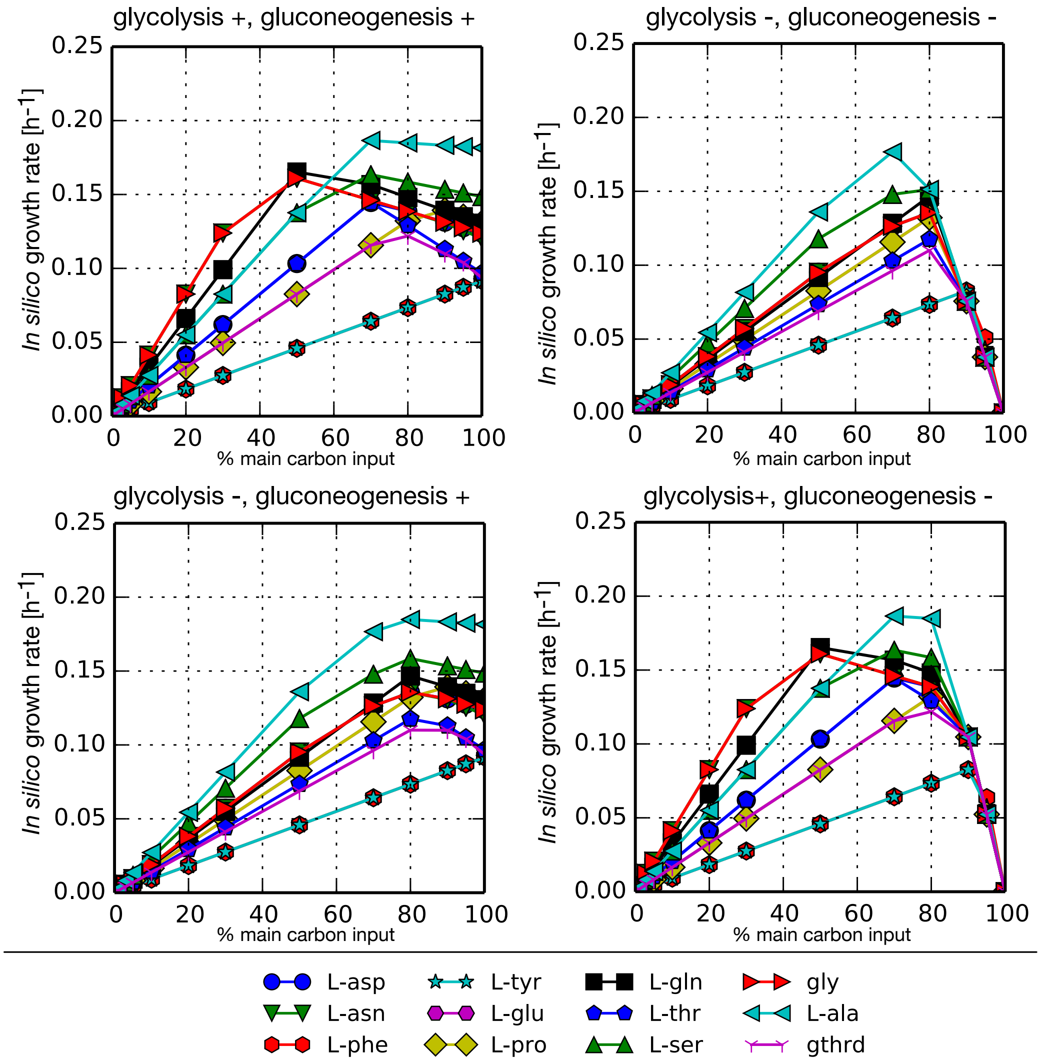

Supplementary Figure 4: *In silico* growth rates for *P. salmonis* LF-89 under different growth scenarios. FBA simulations were performed with model iPS584 using simultaneously two metabolites as main carbon input: glucose and an additional carbon source M (listed below the diagrams). In each simulation maximum carbon uptake was limited to 6 mmol/gCDW-h and it was distributed between glucose and M in different ratios as depicted in each diagram (x axe) where the percentage of total carbon input flux associated to metabolite M is shown. In each simulation metabolite M was also used as the main nitrogen source by blocking the uptake of ammonium. For each (glucose,M) pair four scenarios were analyzed corresponding to combinations of blocked/unblocked (+/-) gluconeogenesis or glycolysis. In all simulations essential metabolites uptake was unconstraint.

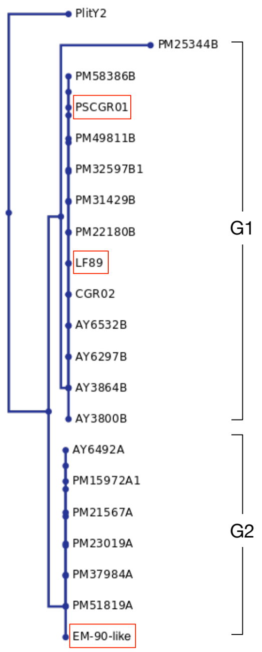

Supplementary Figure 5: Phylogenetic tree of *Piscirickettsia salmonis* strains constructed using their 16S rRNA sequences. The analysis shows that among the three strains used for growth experiments, strains LF-89 and CRG01 belong to the previously reported clade G1 while strain EM-90-like is part of clade G2. The tree was constructed using 20 *P. salmonis* strains and *Piscirickettsia litoralis* Y2 (PlitY2) as an outlier.

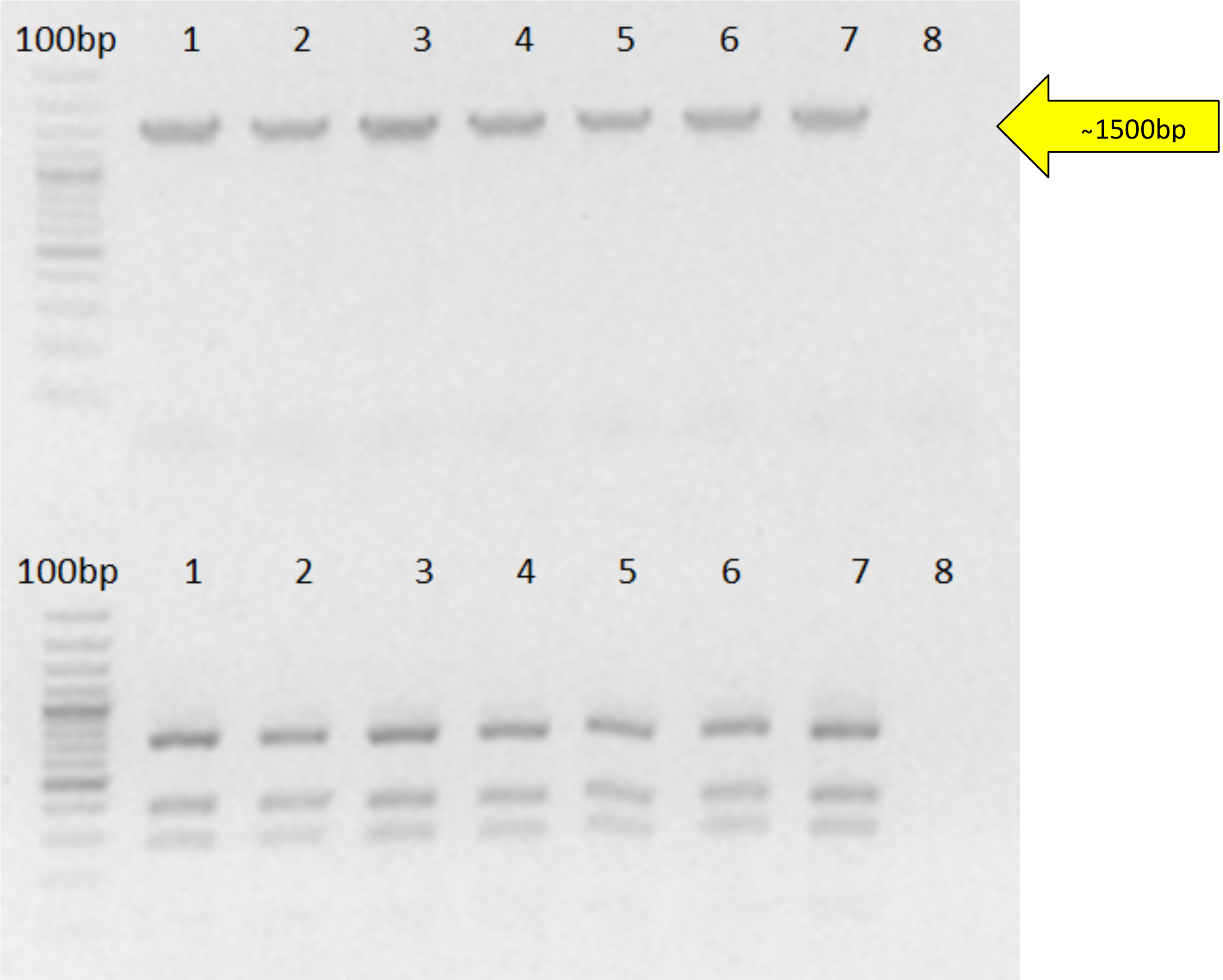

Supplementary Figure 6: Gel electrophoresis. Above: Extracted 16S rDNA from batch cultures of *P. salmonis* LF-89 growing in Basal Medium (lane 1-3), Medium 3: Basal Medium without TCA compounds (lane 4-6), Austral SRS medium supplemented with1 g/L Cysteine, 14mL/L Fetal Bovine Serum, 30 g/L Casein-Peptone Soymeal-Peptone Broth and 15 g/L sodium chloride (lane 7, Positive Control) and water (lane 8, Negative Control). Below: Digested 16S rDNA for the same conditions before mentioned, which results in *P. salmonis*-specific band patterns.

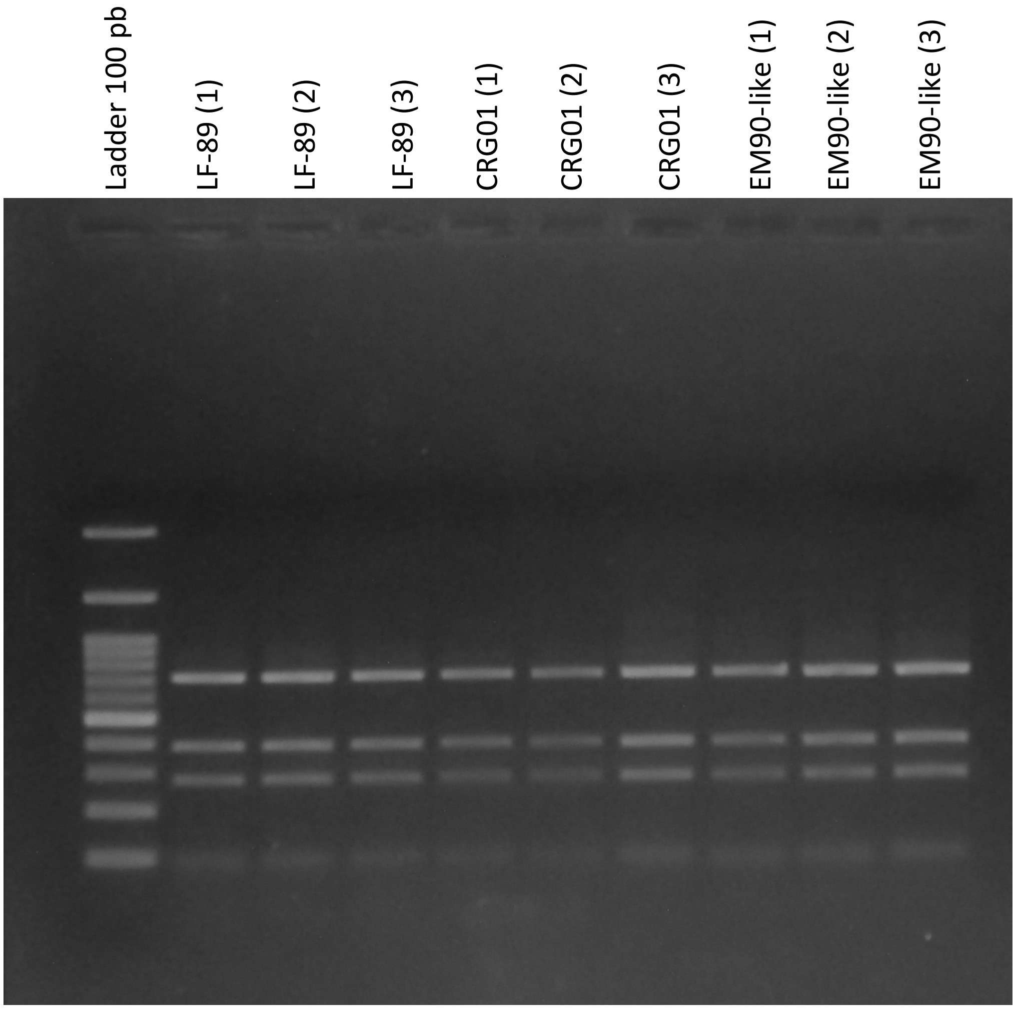

Supplementary Figure 7: Gel electrophoresis. Digested 16S rDNA from additional batch cultures of *P. salmonis* strains LF-89, CRG01 and EM-90-like growing in Medium 3: Basal Medium without TCA compounds. The observed bands agree with *P. salmonis*-specific patterns. Lane 1: 100 bp ruler. Lanes 2-4: LF-89 cultures; lanes 5-7: CRG01 cultures; lanes 8-10: EM90-like cultures.

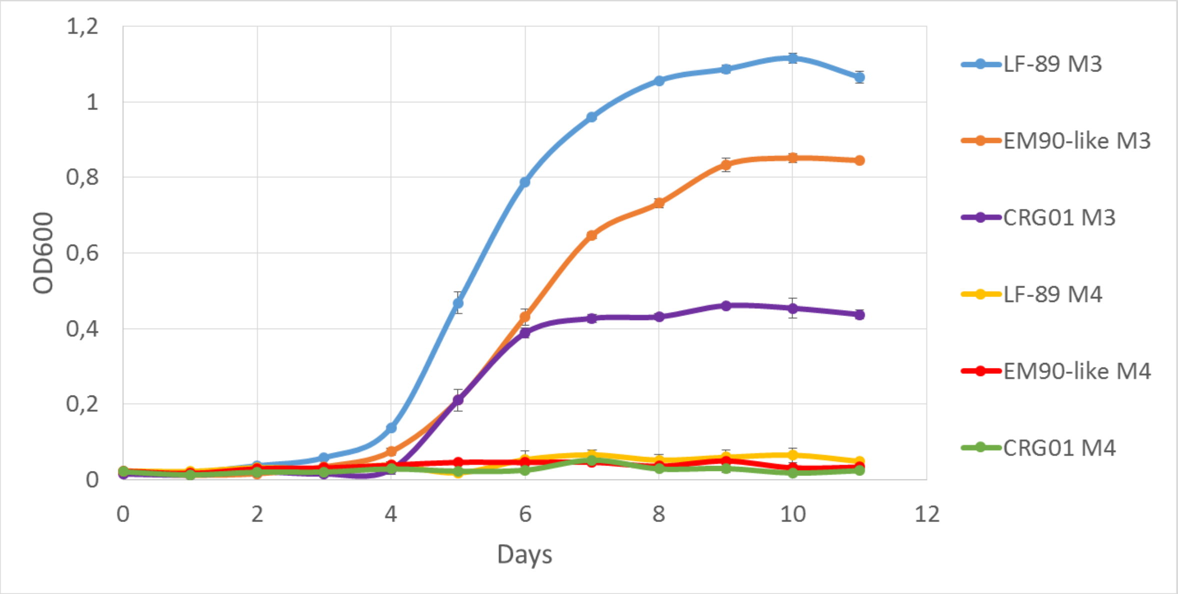

Supplementary Figure 8: Growth curves for *P. salmonis* strains LF-89, CRG01 (clade G1) and strain EM90-like (clade G2) on defined Medium 3 and 4. Medium 3 is basal medium without succinic, malic, fumaric and 2-oxoglutaric acids, i.e., without TCA compounds (BM - TCA). Medium 4 is basal medium without glucose and TCA compounds (BM - GLC - TCA). Full media composition is listed in Table S11 in Supplementary File 2. LF-89 M3: *P. salmonis* LF-89 growing on Medium 3. EM90-like M3: *P. salmonis* EM90-like growing on Medium 3. CRG01 M3: *P. salmonis* CRG01 growing on Medium 3. LF-89 M4: *P. salmonis* LF-89 growing on Medium 4. EM90-like M4: *P. salmonis* EM90-like growing on Medium 4. CRG01 M4: *P. salmonis* CRG01 growing on medium M4.

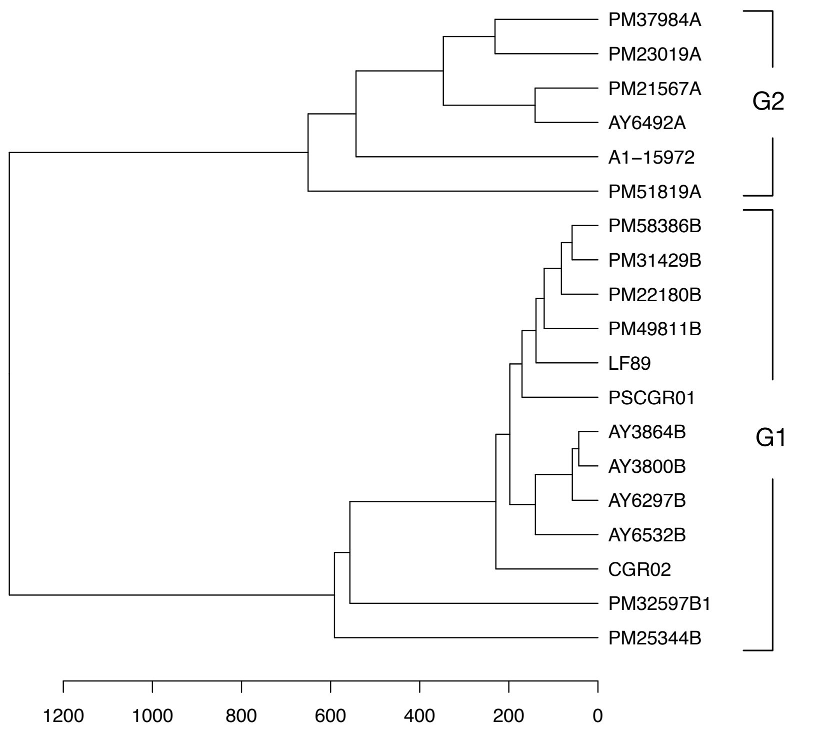

Supplementary Figure 9: Hierarchical clustering of *P. salmonis* strains on their pangenome share and non-shared elements. Two distinct groups can be distinguished among the species strains, consistent with previous findings that place strain LF-89 and A1-15972 in two distinct genotypic groups named G1 and G2

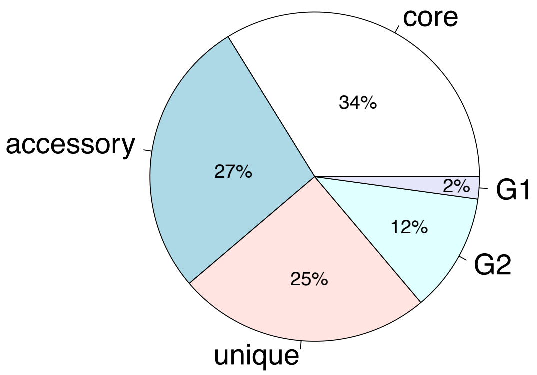

Supplementary Figure 10: *P. salmonis* pangenome. A universe of 4080 orthologs makes *P. salmonis* pangenome. These orthologs can be grouped in the following five categories: *core*: shared among all strains; *G1*: present in two or more strains of the G1 group and absent in G2 strains; *G2*:

present in two or more strains of the G2 group and absent in G1 strains; *unique*: present in a single strain only; and *accessory*: present in some of the strains from groups G1and G2.

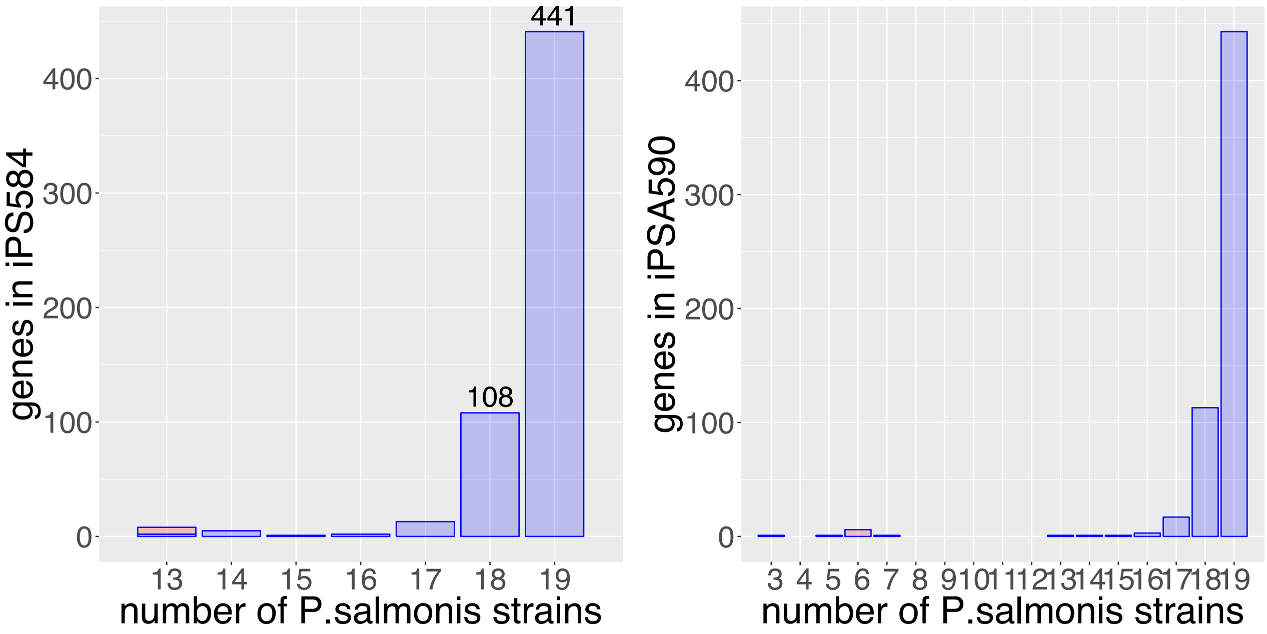

Supplementary Figure 11: Genes in *P. salmonis* metabolic models iPS584 and iPSA590 shared among *P. salmonis* genomes from 19 different strains. In red, genes from model iPS584 that are shared only among all 13 clade G1 strains (left) and genes from model iPS590 shared only among all six clade G2 strains (right).

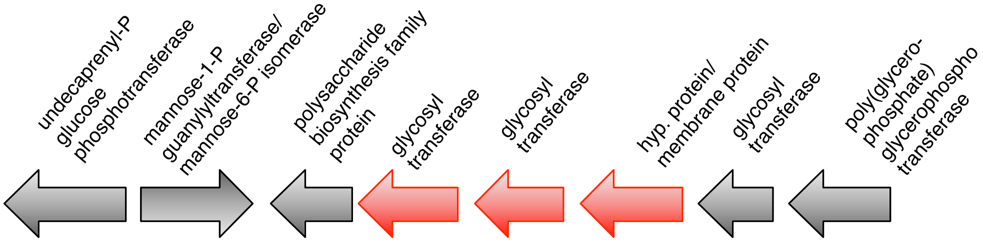

strain/ortholog group	***mcl1516***	***mcl1517***	***mcl1518***	***mcl2497***	***mcl2498***	***mcl2499***	***mcl1519***	***mcl2036***	
LF-89	AKP74764.2	AKP74765.2	AKP74766.1	AKP74767.1	AKP74768.1	AKP74769.1	AKP74770.1	AKP74771.1	
AY3800B	AOS36249.1	AOS36248.1	AOS36247.1	AOS36246.1	AOS36245.1	AOS36244.1	AOS36243.1	AOS36242.1	
AY3864B	APS80963.1	APS80962.1	APS80961.1	APS80960.1	APS80959.1	APS80958.1	APS80957.1	APS80956.1	
AY6297B	APS67950.1	APS67949.1	APS67948.1	APS67947.1	APS67946.1	APS67945.1	APS67944.1	APS67943.1	
AY6532B	APS69435.1	APS69436.1	APS69437.1	APS69438.1	APS69439.1	APS69440.1	APS69441.1	APS69442.1	
CGR02	AMA41060.1	AMA41059.1	AMA41058.1	AMA41057.1	AMA41056.1	AMA41055.1	AMA41054.1	AMA41053.1	
PM22180B	APS72224.1	APS72223.1	APS72222.1	APS72221.1	APS72220.1	APS72219.1	APS72218.1	APS72217.1	G1
PM25344B	APS82006.1	APS82005.1	APS82004.1	APS82003.1	APS82002.1	APS82001.1	APS81999.1	-	
PM31429B	APS77186.1	APS77185.1	APS77184.1	APS77183.1	APS77182.1	APS77181.1	APS77180.1	APS77179.1	
PM32597B1	ALB21304.1	ALB21303.1	ALB21302.1	ALB21301.1	ALB21300.1	ALB21299.1	ALB21298.1	ALB21297.1	
PM49811B	APS60941.1	APS60940.1	APS60939.1	APS60938.1	APS60937.1	APS60936.1	APS60935.1	APS60934.1	
PM58386B	APS64179.1	APS64178.1	APS64177.1	APS64176.1	APS64175.1	APS64174.1	APS64173.1	APS64172.1	
PSCGR01	ALY01547.1	ALY01546.1	ALY01545.1	ALY01544.1	ALY01543.1	ALY01542.1	ALY01541.1	ALY01540.1	

	mcl1516	***mcl1517***	***mcl1518***	***mcl3131***	***mcl3132***	***mcl3133***	***mcl1519***	***mcl2036***	
A1-15972	ALA26418.1	ALA26419.1	ALA26420.1	ALA26421.1	ALA26422.1	ALA26423.1	ALA26424.1	ALA26425.1	
PM21567A	APS47197.1	APS47198.1	APS47199.1	APS47200.1	APS47201.1	APS47202.1	APS47203.1	APS47204.1	
PM51819A	APS57660.1	APS57659.1	APS57658.1	APS57657.1	APS57656.1	APS57655.1	APS57654.1	APS57653.1	G2
PM37984A	APS54572.1	APS54571.1	APS54570.1	APS54569.1	APS54568.1	APS54567.1	APS54566.1	APS54565.1	
AY6492A	APS43843.1	APS43844.1	APS43845.1	APS43846.1	APS43847.1	APS43848.1	APS43849.1	APS43850.1	
PM23019A	APS51363.1	APS51362.1	APS51361.1	APS51360.1	APS51359.1	APS51358.1	APS51357.1	APS51356.1	

Supplementary Figure 12: Gene clusters in *P. salmonis* strains genomes involved in lipopolysaccharides metabolism. Two similar gene clusters are found in G1 and G2 strains. However, G1 and G2 strains have different genes for putative glycosyltransferases in these clusters (red arrows).
